# Bacterial DNA involvement in carcinogenesis

**DOI:** 10.3389/fcimb.2022.996778

**Published:** 2022-10-12

**Authors:** Han Shuwen

**Affiliations:** ^1^ Huzhou Central Hospital, Affiliated Central Hospital Huzhou University, Huzhou, China; ^2^ Graduate School of Medical college of Zhejiang University, Hangzhou, China; ^3^ Key Laboratory of Multiomics Research and Clinical Transformation of Digestive Cancer, Huzhou, China

**Keywords:** bacterial DNA, human genome, gene integration, cancerogenesis, hypothesis

## Abstract

The incidence of cancer is high worldwide, and biological factors such as viruses and bacteria play an important role in the occurrence of cancer. *Helicobacter pylori*, *human papillomavirus*, *hepatitis B viruses* and other organisms have been identified as carcinogens. Cancer is a disease driven by the accumulation of genome changes. Viruses can directly cause cancer by changing the genetic composition of the human body, such as cervical cancer caused by *human papillomavirus* DNA integration and liver cancer caused by *hepatitis B virus* DNA integration. Recently, bacterial DNA has been found around cancers such as pancreatic cancer, breast cancer and colorectal cancer, and the idea that bacterial genes can also be integrated into the human genome has become a hot topic. In the present paper, we reviewed the latest phenomenon and specific integration mechanism of bacterial DNA into the human genome. Based on these findings, we also suggest three sources of bacterial DNA in cancers: bacterial DNA around human tissues, free bacterial DNA in bacteremia or sepsis, and endogenous bacterial DNA in the human genome. Clarifying the theory that bacterial DNA integrates into the human genome can provide a new perspective for cancer prevention and treatment.

## 1 Introduction

According to the statistics of the World Health Organization, it is estimated that there will be 19.3 million new cancer cases and nearly 10 million cancer deaths in 2020 around the world (Sung et al., 2021). Assuming that the estimated global incidence rate in 2020 remains unchanged, there will be 28.4 million new cancer cases in 2040, an increase of 47% over the corresponding 19.3 million cases in 2020 (Sung et al., 2021). The consequences are not optimistic. The occurrence of cancer is caused by the interaction between genetic factors and carcinogenic factors in the external environment. Among the carcinogenic factors, there are many physical and chemical factors, such as ultraviolet rays (Rivas et al., 2020) and nitrosamines (Liu et al., 2021). Another factor is the biological factor that causes cancer, that is, infection caused by microorganisms, viruses and bacteria (De Martel et al., 2017; Watanabe et al., 2021). Using metagenome sequencing, 16S rRNA detection and other technologies, the microbial community composition of various cancers, such as colon cancer, pancreatic cancer, breast cancer, and lung cancer, was studied and identified, and the existence of bacteria, viruses and other microorganisms was found (Nejman et al., 2020). At present, the role of specific microorganisms in the pathogenesis of cancer has been widely studied. *Helicobacter pylori* (*H. pylori*) (Ford et al., 2022), *human papillomavirus* (HPV) (Rebolj et al., 2022), and *hepatitis B viruses* (HBV) (Kamiza et al., 2022) are considered the main infection sources of 1.9 million new cancer cases worldwide every year. These cancer-causing microorganisms can lead to carcinogenesis by triggering inflammation (Chung et al., 2018), releasing metabolites (Yang et al., 2022), inducing DNA damage (Wang et al., 2016) and changing the cancer immune microenvironment (Zegarra-Ruiz et al., 2021).

Cancer is a disease driven by the accumulation of genome changes. The essence of cancer is always genetic changes. The methods of gene change include gene mutation (Greathouse et al., 2018), gene integration (Sung et al., 2012), gene repair damage (Imai et al., 2021), etc. Nonlong terminal repeats, such as long interspersed elements (LINEs) (Lander et al., 2001), and mobile elements, such as Alu elements (Steely et al., 2021), can jump actively in the human genome and induce cancers by causing gene mutations. For example, LINE-1 is inserted into the adenomatous polyposis coli (APC) gene of colon cancer (Scott et al., 2016), and Alu is inserted into mixed-lineage leukemia (MLL) genes (Stumpel et al., 2013). The Whitehead Institute of Biomedicine found that even a single base insertion in some genes may change the original operon structure and then have a certain impact on the functions of related genes. For example, the formation of the LIM domain only 2 (LMO2) oncogene expression enhancer caused by insertion mutation transforms somatic cells into primary leukemia cancer cells (Abraham et al., 2017). The mismatch repair gene mutL homolog 1 (MLH1) and mismatch repair gene mutL homolog 2 (MSH2) genes are related to hereditary nonpolyposis colorectal cancer syndrome (HNPCC) (Bouvet et al., 2019). MLH1 can induce a leukemia phenotype by changing the protein structure and inactivating inherent MLL1 function (Whitman et al., 2005).

Viruses can integrate into the human genome and cause cancer-related mutations. The integration of HPV is the key event leading to HPV-related cancers. Up to 80-100% of cervical cancers have HPV16 or HPV18 integration (Corden et al., 1999; Melsheimer et al., 2004). There are more than 1500 integration sites reported in the literature, such as 3q28, 8q24.21, 13q22.1, 2q22.3, 3p14.2, 8q24.22, 14q24.1, 17p11.1, 17q23.1, and 17q23.2 (Bodelon et al., 2016). When HPV integration leads to the deletion of E1 and E2 proteins, cell proliferation increases aberrantly (Pokrývková et al., 2019; Hirose et al., 2020). The integration of HBV DNA into the host hepatocyte genome is key to hepatocellular carcinoma (HCC) (Sung et al., 2012). Telomerase reverse transcriptase (TERT) (Sze et al., 2021), MLL4 (Saigo et al., 2008) and cyclin E1 (CCNE1) (Bayard et al., 2018) are common insertion genes for HBV integration. A region from nt1100 to nt1500 containing multiple HCC risk mutation sites (OR > 1) was identified as a potential HCC-related mutational hot zone (Zheng et al., 2021). The chromosome change rate and p53 mutation inactivation rate of HBV-related cancers are high (Song et al., 2021), leading to the activation of the mTOR signaling pathway (Luo et al., 2021) or WNT/β-catenin pathway (Dang et al., 2019), thus increasing the proliferation of cancer cells.

**Figure 1:**
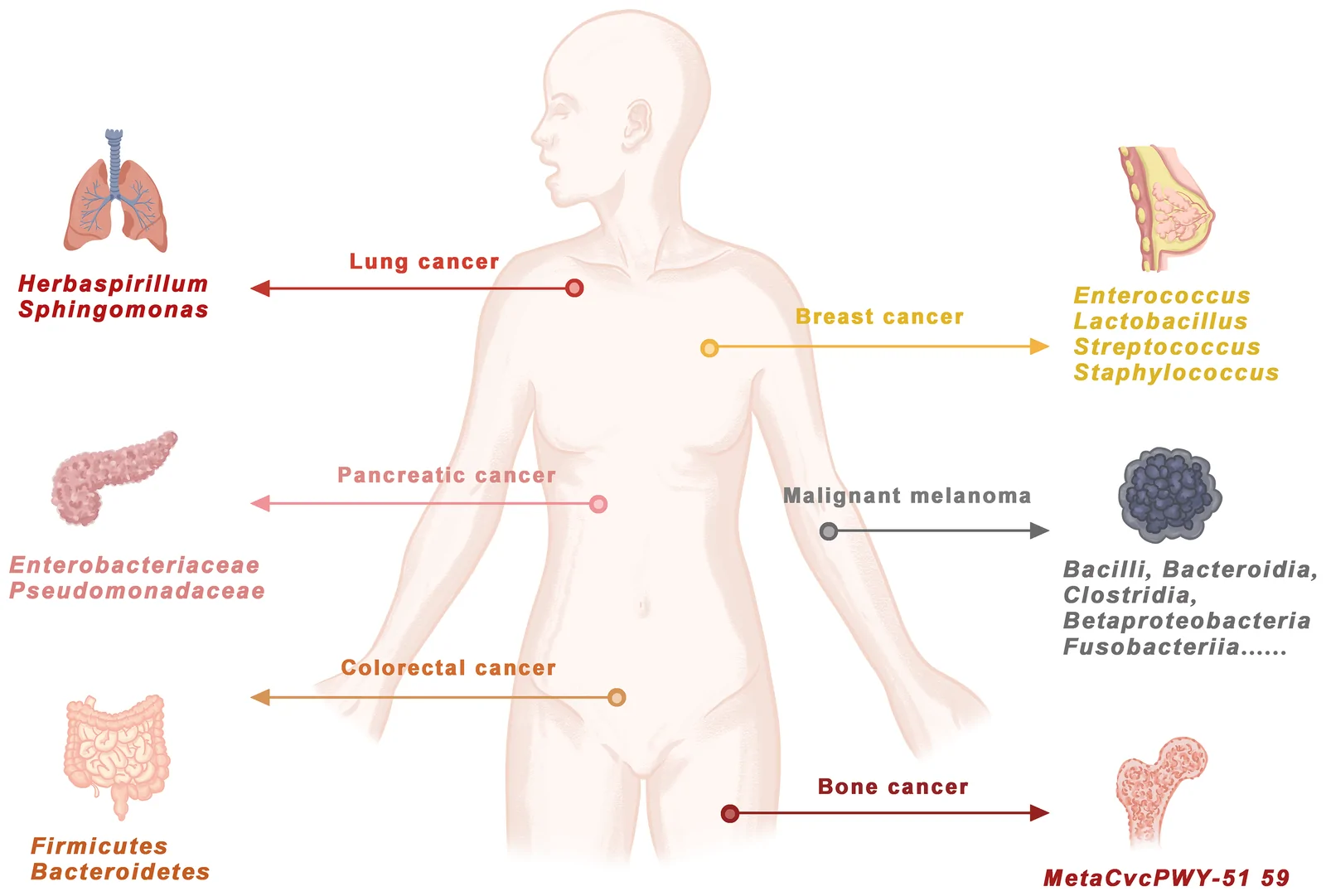
Bacterial DNA exists in human cancers.Through genome sequencing, PCR amplification, 16S RNA detection and KEGG/TCGA database comparison, bacterial DNA was confirmed in lung cancer, pancreatic cancer, breast cancer, malignant melanoma, colorectal cancer and bone cancer. DOI: 10.1126/science.aay9189.

The number of bacteria in a healthy adult is only one-tenth the number of viruses. However, bacteria should not be underestimated. In the human body, there are more bacterial cells than human cells (Sender et al., 2016). Bacteria have been neglected in cancer research because of their extremely low biomass (Salter et al., 2014; Eisenhofer et al., 2019). Recently, next-generation sequencing methods for microbiome research have become more sensitive than ever to study microbial communities, their genomes and their functions. Bacterial DNA from 1010 tumor samples and 516 normal samples from 7 cancer types (Nejman et al., 2020) was quantitatively analyzed by 16S rDNA sequencing. Some bacteria were found to accumulate in the cytoplasm, and they were called intracellular bacteria. These bacteria might be cancer specific. Different cancer tissues harbor different bacteria (**Figure 1**). Therefore, this article will review the latest phenomena related to bacteria and cancer and the specific integration mechanism of bacterial DNA. The integration of bacterial DNA into the human genome may act as a cis-element to affect the function of host genes, activate proto-oncogenes, silence tumor suppressor genes and regulate cancer-related pathways. The integration of bacterial DNA is an important mechanism of carcinogenesis, which can provide theoretical support for the search for new cancer diagnostic and therapeutic targets.

## 2 Research of bacteria involved in human cancer

The composition of bacteria surrounding cancer tissues is different from that in normal tissues. Recently, many studies have shown that human lungs contain various microbiota closely related to the development of lung cancer. The ecological diversity in noncancerous (immediate autopsy and hospital biopsy) tissues, noncancerous adjacent tissues and cancer tissue samples was detected by 16S rRNA gene sequencing. At the phylum level, *Proteobacteria* (Kruskal−Wallis P = 0.0002) was increased, and *Firmicutes* (Kruskal−Wallis P = 0.04) was decreased in hospital lung biopsies, as well as in cancer tissues, from the National Cancer Institute-Maryland (NCI-MD) study (Huang et al., 2011). In The Cancer Genome Atlas (TCGA) study, a similar increase in *Proteobacteria* (Mann−Whitney P = 0.02) between noncancer lung tissues and lung cancer was also observed. This finding indicated that this was a recurring phenomenon in lung cancer (Huang et al., 2011). In the NCI-MD study, 32 significantly different genera in lung squamous cell carcinoma (SCC) (n = 47) and lung adenocarcinoma (AD) (n = 67) (Student’s t test; MW P < 0.05) were found. Nine of them, including *Acidovorax*, *Brevundimonas*, *Comamonas*, *Tempiddimonas*, *Rhodoferax*, *Klebsiella*, *Leptospira*, *Polaromonas* and *anaerobes*, were still significant after multiple testing correction (FDR) (Greathouse et al., 2018). These same observations were validated in the TCGA dataset (AD = 485, SCC = 489) (Mann−Whitney FDR corrected P value < 0.05). Adjusted logistic regression analysis was performed in each group, and 6/9 genera were still significantly associated with increased odds of SCC compared with AD. Research on whether these cancer-related genera would change after quitting was performed, and the results showed that *Acidobacteria*, *Klebsiella*, *Tepidimonas*, *Rhodoferax* and *anaerobes* remained significant (Greathouse et al., 2018).

There are thousands of microorganisms in the intestinal tract. The microbial composition of colorectal cancer (CRC) patients is significantly different from that of healthy individuals. Bacteria invading the mucous layer of the colon axis in every FAP patient were detected by 16S rRNA gene analysis and fluorescence *in situ* hybridization (FISH) probe labeling polyps and macroscopic normal tissues. The main biofilm members were identified as *Escherichia coli* (*E. coli*) and *Bacteroides fragilis* (*B. fragilis*). The intestinal epithelial cells of all CRC patients with bacterial biofilms in their intestines were invaded by biofilm community members. This finding was similar to those of sporadic colorectal cancer patients (Dejea et al., 2018).. Compared with healthy subjects (22% pks+*E. coli* and 30% *B. fragilis*), pks+*E. coli* (68%) and *B. fragilis* (60%) in the mucosa were increased significantly in the mucosa of familial adenomatous polyposis (FAP) patients. Pks+*E. coli* and *B. fragilis* were not associated with polyps or normal mucosa in FAP patients. The polymerase chain reaction (PCR) analysis showed that the mucosal biofilm of early FAP patients contained clbB and BFT corresponding to the carcinogenic subtypes of *E. coli* and *B. fragilis*, respectively. The proteins existed in the mucous layer, which was in direct contact with the FAP epithelium (Dejea et al., 2018). Quantitative polymerase chain reaction (qPCR) analysis showed that 9 cases (82%) of 11 quick-frozen primary cancers were positive for *Fusobacterium*. *Fusobacterium* could be isolated from 73% of cancers (n = 8/11). *Fusobacterium* could also be isolated from liver metastatic sites of patients with liver metastases from CRC. *Fusobacterium nucleatum* (*F. nucleatum*) was highly enriched in CRC tissues and migrated with the spread of CRC cells. Many studies have reported that *F. nucleatum* supports the growth and development of cancers (Yang et al., 2017; Guo et al., 2020; Hong et al., 2021). *F. nucleatum* increases the incidence of intestinal cancers by creating a more suitable microenvironment for cancer growth. Colon cancer cells treated with *F. nucleatum* had stronger division, proliferation and invasion abilities. Inhibiting the growth of *F. nucleatum* might inhibit the development of CRC. Based on gut microbiome sequencing, some researchers constructed CRC screening models. Twenty microbial markers for distinguishing CRC patients from healthy controls through metagenomic sequencing of fecal samples were identified (Yu et al., 2017). Similarly, an early detection model to distinguish colorectal adenoma from healthy individuals and an early detection model to distinguish adenoma from CRC were established using oral microbial markers (Zhang et al., 2020).

Pancreatic cystic lesions, including intraductal papilloma (IPMNs), are common. They are known as precursors of pancreatic cancer. The adhesion of bacteria to epithelial cells and their ability to live in epithelial cells are important factors to exert their virulence. Healthy pancreatic cells (hTERT-HPNE), early differentiated pancreatic cancer cells (Capan-2) and late differentiated pancreatic cancer cells (AsPC-1) were incubated with a single strain for two hours. This short-term coculture with pancreatic cells allowed most isolated bacteria to enter human pancreatic cells and survive (Geller et al., 2017). In all pancreatic cells, the top survivors were *Enterobacter cloacae*, *Enterococcus faecalis*, *Enterococcus faecalis* and *Klebsiella pneumoniae*. Halimi A showed that bacteria from IPMN capsule fluid could invade and survive in pancreatic cells *in vitro*. Cyst is a potential microbial pool that continuously provides microorganisms for healthy and cancerous pancreatic cells (Halimi et al., 2021). Bacterial DNA from 65 pancreatic cancer patients was sequenced by 16S rDNA and PCR. The results showed the presence of bacterial DNA in pancreatic cancer cells (Geller et al., 2017). Compared with the bacterial DNA in the KEGG library, the most common species in pancreatic cancer cells was *γ-proteobacteria*, accounting for 51.7% of the total reads. Most of them were members of *Enterobacteriaceae* and *Pseudomonas*. The presence of numerous *proteobacteria* in the duodenum with open pancreatic ducts suggested that retrograde migration of bacteria from the duodenum to the pancreas might be the source of bacteria associated with pancreatic duct adenocarcinoma (PDAC) (Geller et al., 2017).

Forty-one different bacteria from melanoma samples were identified by the Weizmann Institute of Science in Israel through 16S rRNA gene sequencing (Kalaora et al., 2021). Bacteria including *Bacilli*, *Bacillus*, *Clostridia*, *Betaprotebacter*, *Fusobacteria*, *Gammaproteobacteria*, *Actinobacteria*, and *Alphabetobacteria* showed high abundance in melanoma cells. The low passage cell line from the same melanoma cell was cocultured with representative intracellular bacteria, and the entry of bacteria into cells was detected by FISH. For example, 51AL melanoma cells and 55A3 melanoma cells were cocultured with *Staphylococcus carinii* and *Actinomycetes dentatum*. The presence of bacteria in melanoma cells was confirmed by immunofluorescence staining of 51AL and 55A3 cells with lipoteichoic acid. *F. nucleatum* and *Actinomycetes* were labeled by copper-free chemicals, and the labeled bacteria were cocultured with 51AL and 55A3 cells. Correlative light and electron microscopy (CLEM) analysis of cells cocultured with *F. nucleatum* and *Actinomycetes* confirmed that these bacteria entered melanoma cells (Kalaora et al., 2021). Subsequently, HLA peptidyomics was used to identify peptide libraries derived from intracellular bacteria that had been present on HLA-I and HLA-II molecules in melanoma cancers. Analysis of 17 melanoma metastases (from 9 patients) revealed 248 unique HLA-I and 35 unique HLA-II peptides from 248 bacteria. Recurrent bacterial peptides in tumors of different patients and in different tumors of the same patient were both identified (Kalaora et al., 2021).

Fresh frozen breast specimens from 221 breast cancer patients and 87 nonbreast cancer patients were analyzed. At the genus level, *Pseudomonas* accounted for a higher proportion of the breast microbiome in cancer tissues than in other tissues, while *Proteus* was the second most abundant genus in cancer tissues and was largely absent in nontumor tissues. *Porphyromonas* and *Azomonas* were also more abundant in cancer tissues than in other tissues. In particular, *Porphyromonas*, *Lacibacter*, *Ezakiella*, and *Fusobacterium* were more abundant in high-stage cancers than in low-stage cancers (Tzeng et al., 2021). Cancer tissues, adjacent tissues and lymph node tissues from paired breast cancer patients were collected, and qPCR combined with 16S sequencing was used to analyze the composition of their respective microbiota. Gram-positive bacteria were the main types, with *Enterococcus*, *Lactobacillus*, *Streptococcus* and *Staphylococcus* accounting for approximately 80% (Fu et al., 2022). Bacteria existing in the cytoplasm were observed by high-resolution electron microscopy. After clearing these bacteria with an A cell-permeable antibiotic (doxycycline), we found that the cancer tissue weight was not affected, but the lung metastasis decreased significantly. 16S RNA sequencing analysis was performed on *in situ* tumors, visible lung metastases, lung tissues with tiny metastases and normal lung and breast tissues. The results suggested that bacteria with early lung metastases might still bear the microbiota characteristics of cancer tissues in situ.

Circulating tumor cells carrying intracellular bacteria to distal organs were found by isolating and staining circulating cancer cells. *Streptococcus*, *Lactobacillus*, *Staphylococcus* and other bacteria can invade cancer cells and reshape the cytoskeleton. When metastatic cancer cells invade the circulatory system, they need to undergo fluid shear stress, which often triggers apoptosis. By placing tumor cells containing bacteria and those without bacteria into the circulation system with a peristaltic pump, which can simulate the shear stress of fluid in blood vessels, cells containing bacteria had a higher survival rate than cells without bacteria. When the living tumor cells containing bacteria and not containing bacteria were spread on the plate, the living cells with bacteria had better adhesion ability and a larger cytoskeleton. This phenotype indicated that bacteria might play a role in the actin cytoskeleton. RhoA, Rac and Cdc42 are involved in the regulation of the actin cytoskeleton in cells. Fluorescence resonance energy transfer (FRET) sensor analysis of activated RhoA and Western blot analysis of RhoA-GTP showed that the invasion of bacteria indeed suppressed RhoA and ROCK activation. The inhibition of ROCK kinase (downstream of RhoA) could also eliminate the difference in viability caused by bacterial invasion. Bacteria can help transform breast cancer cells, prevent them from being damaged in the process of metastasis, and help them transfer to distant organs in the body (Fu et al., 2022).

Infection with *H. pylori* and a family history of gastric cancer are the main risk factors for gastric cancer (Lee et al., 2016). Approximately three thousand first-degree relatives of patients with gastric cancer were screened, and 1838 participants with *H. pylori* infection were randomly assigned to receive eradication therapy (lansoprazole [30 mg], amoxicillin [1000 mg], clarithromycin [500 mg], twice daily for 7 days) or placebo. During a median follow-up of 9.2 years, gastric cancer developed in 10 participants (1.2%) in the treatment group and in 23 participants (2.7%) in the placebo group (hazard ratio, 0.45; 95% confidence interval [CI], 0.21 to 0.94; log-rank test P = 0.03). Gastric cancer developed in 0.8% (5/608) of participants with eradication of *H. pylori* infection and developed in 2.9% (28/979) of participants with persistent infection (hazard ratio, 0.27; 95% CI, 0.10 to 0.70). It was more common in the treatment group than in the placebo group (53.0% vs. 19.1%; P < 0.001). In patients with *H. pylori* infection whose first-degree relatives had a family history of gastric cancer, *H. pylori* eradication therapy could significantly reduce the risk of gastric cancer (Choi et al., 2020).

## 3 Research on bacterial DNA in the human cancer genome

In 2001, an important paper based on the sequencing results of the human genome proposed that some human genes may come from horizontal gene transfer of bacteria by comparing the genomes of bacteria and some animals. However, those ideas were quickly countered. Blood, liver and three types of adipose tissue samples from 40 subjects were studied recently. Bacterial genetic materials in each tissue sample were found, and the types of bacteria and the amount of bacterial DNA were different. In addition, DNA in the tissue samples was detected not only from gut bacteria but also from those commonly found in soil or water. This finding implied that viscera may be regularly exposed to exogenous genetic material (Anhê et al., 2020).

In 2020, the microbial composition of seven cancers, including breast cancer, lung cancer, ovarian cancer, pancreatic cancer, melanoma, bone cancer and brain cancer, was revealed by comparing thousands of cancer tissues with nearby normal tissues. Microbes were found in every cancer tissue from the head to the bone, and the microbe composition in every cancer tissue was different. Among them, breast cancer had the highest diversity, with 16.4 bacteria in each breast tumor sample, while other tumors had only 9 species on average (Nejman et al., 2020). Cancer tissues were found to contain bacteria in the past, but the content was very low. Some researchers think this might be the result of sample contamination. Various methods have been adopted to avoid contamination. For example, to address possible contamination of samples between storage and the laboratory, they took more than 100 sections from each paraffin block edge of paraffin embedded samples (no sample tissue) as a control group. In total, 1010 tumor samples and 516 normal tissue samples were analyzed. Bacterial DNA levels in samples from each type of cancer tissue were significantly higher than those in normal tissues and paraffin sections. Among the seven tumors, melanoma had the lowest bacterial DNA positive rate (14.3%), while breast cancer, pancreatic cancer and bone cancer had higher positive rates, all exceeding 60%. The other 400 cancer specimens were detected by immunohistochemical methods using bacterial lipopolysaccharide and lipoteichoic acid antibodies (specific components of the cell walls of Gram-negative bacteria and Gram-positive bacteria, respectively), and all types of cancers had positive results. The bacterial RNA in 400 cancer specimens was detected by FISH, and the results were also positive. Bacteria mainly exist in cancer cells and immune cells, especially in their cytoplasm. Using an optical microscope, researchers observed that most of them were located in the cytoplasm near the nuclear membrane of the nucleus, and many of them had no cell walls (Nejman et al., 2020).

Paired RNA sequencing data from patients with chronic lymphocytic leukemia and healthy donors were analyzed to look for incorporation of bacterial DNA into the human somatic cell genome (Riley et al., 2013). The Burrows−Wheeler Comparator (BWA) was applied first to the human genome and then to the bacterial genome reference. Mapping sequencing reads of the human genome, microbiome, and bacteria were distinguished, and all reads with alignment coverage less than 90% and low-complexity reads containing long poly-A/-T/-g/-C sequences were excluded. More bacterial DNA was found in chronic lymphocytic leukemia specimens, and the bacterial genera integrated in the whole human genome were Pseudomonas sp., Mesorhizobium sp., and Acinetobacter sp. (unspecified sites) (Sun and Ma, 2019). Most bacterial integration events (36%) occurred in the intronic region, 22% in the exon region, and 17% in the 3’ UTR region. Genes such as SNORD141A/B, MIR4507, MALAT1, CD74, HLA-B, LGT, and HLA-C are integration hotspots (Jima et al., 2010; Riley et al., 2013; Sun and Ma, 2019; Akimova et al., 2022). Data published by the Thousand Genomes Project were analyzed, and more than 7,000 cases of horizontal gene transfer from bacteria were found. After analyzing TCGA sequences, the researchers found 691,000 horizontal gene transfers, 99.9 percent of which came from cancer samples. One-third of the bacterial sequences in the entire human genome came from Acinetobacter and were inserted into the mitochondrial genome (Hancks and Kazazian, 2012; Riley et al., 2013). Mitochondrial genomes or nuclear mitochondria of acute myeloid leukemia samples are the most common Acinetobacter-like DNA integrations (1000 Genomes Project Consortium et al., 2010). The integration of Pseudomonas DNA in gastric adenocarcinoma samples was mainly concentrated in five genes. Among the 5 genes, 4 were upregulated proto-oncogenes, including TMSB10, CEACAM5, CEACAM6 and CD74 (Han et al., 2008; 1000 Genomes Project Consortium et al., 2010; Gold et al., 2010; Ren et al., 2020).

If bacterial integration is an important mechanism of carcinogenesis, we hope that further studies in this area will reveal similar integration from specific mobile elements and viruses, including the insertion and inactivation of coding genes and the transfer of oncogenic proteins and peptides. Is bacterial DNA integration similar in all cancers, or does each cancer have its own specific bacterial DNA integration pathway? What factors contribute to bacterial integration sites in each cancer? Which bacterial DNA integration sites are consistent? Many mechanistic issues remain to be further studied.

## 4 Potential mechanisms of incorporation of bacterial DNA into human DNA

### 4.1 Bacterial DNA released from the bacteria

Natural transformation is a process by which bacteria spontaneously absorb, integrate and excrete foreign DNA (Johnston et al., 2014). This process is the source of bacterial genetic diversity and evolutionary flexibility. Many bacterial genera release their own DNA into the extracellular environment by secretion or autolysis. This behavior provides conditions for DNA uptake by other cells. DNA release from bacterial cells can be directed to selection, mainly cell division and replication (vertical gene transfer) and direct investment in the replication of genetic information in other cells (horizontal gene transfer) (Mao et al., 2014). DNA release can be mediated by secretion, for example, through the type IV secretion system and membrane capsules or by autolysis of a subset of the population (Li et al., 2005; Ibáñez de Aldecoa et al., 2017). The cagI-pathogenic island (cagPAI) of *H. pylori* contains 32 genes encoding the type IV secretion system (T4SS) and CagA protein (Wroblewski and Peek, 2016; Jia et al., 2018). Intraspecific variation of DNA release was found in Neisseria gonorrhoeae (mediated by both lysis and secretion) (Zaremba et al., 2014). In addition, wild-type Bacillus subtilis strains release free DNA in a lyse-independent manner (Cañas et al., 2011). The analysis of DNA release among different strains was relatively simple, and these data indicated the involvement of cells in DNA release and DNA uptake.

### 4.2 Bacterial DNA entering host cells

It is common for bacteria to transfer horizontally because the DNA of bacteria is in the cytoplasm. The probability of a piece of DNA being integrated into the bacterial genome is high if it can pass through the cell wall and cell membrane of bacteria. Bacterial cells were added to the mammalian cell line medium. After several days of cultivation, one out of every 10,000 mammalian cells was positive for bacterial plasmids. After adding an enzyme that can digest DNA outside the cell membrane and remove the cell membrane, bacterial genes could still be detected in mammalian cell lines. This confirmed that bacterial DNA could enter human cells (Waters, 2001). There is a second barrier in the genome of eukaryotic cells: the nucleus. Usually, DNA is tightly condensed in chromosomes, which restricts the splicing of foreign DNA fragments into the genome. DNA generally cannot exit the nucleus because it is a D+ protein, which is too large to pass through the nuclear pore (ignore nuclear pore identification first). Bacterial genetic material, however, is relatively small, and it is relatively easy to enter and leave the nucleus. The transmembrane transport protein complex on the nucleus is called the nuclear pore complex, which is a bifunctional and bidirectional hydrophilic nucleocytoplasmic exchange channel (Kim et al., 2018). Bacterial DNA can interact with nuclear compounds and enter the nucleus of host cells through nuclear pores. In the process of bacterial pathogenicity, the bacterial type IV secretion system (T4SS) mediates the DNA transfer of cell-to-cell binding and the cross-border protein transfer to eukaryotic host cells (Schröder et al., 2011). *Bartonella henselae* can transfer its cryptic plasmid into the human endothelial cell line EA.hy926 by its T4SS VirB/VirD4. DNA transfer to EA.hy926 cells were demonstrated by the insertion of reporter gene derivatives of *Bartonella*-specific mobile plasmids generated by the eukaryotic EGFP expression cassette. Egfp gene expression in EA.hy926 cells required cell division, suggesting that nuclear envelope rupture might facilitate passive entry of transferred ssDNA into the nucleus, a prerequisite for EGFP gene synthesis of complementary strands and transcription. During human infection with *Bartonella*, T4SS-dependent DNA might be naturally transferred into host cells (Schröder et al., 2011).

### 4.3 Bacterial induction of host genome damage

#### 4.3.1 Bacterial induction of host DNA double-strand breaks

Many bacteria can cause single-strand breaks (SSBs) or double-strand breaks (DSBs) of human DNA (Pleguezuelos-Manzano et al., 2020; Imai et al., 2021). *E. coli* carries pathogenic PKS islands and encodes an enzyme that binds to Colibactin3. Colibactin3 is thought to alkylate DNA on adenine and induce DSBs in cultured cells (Imai et al., 2021). Genetically toxic pks+ *E.* coli were repeatedly injected into healthy human intestinal organs. Immunostaining confirmed that the pks+ *E. coli*-like organ model could cause double-strand cross-linking and DNA double-strand damage. Under the intervention of pks+ *E. coli*, the single base substitution ratio of cells increased significantly. In addition, colibactin could bind two adenines simultaneously and cross-link them. By analyzing Dutch samples from 3668 different cancer types, DNA mutation patterns caused by colibactin were found in various cancers and was more common in CRC than in other types of cancer (Pleguezuelos-Manzano et al., 2020). After, 2208 CRC samples from 100,000 genome projects in the UK were analyzed, and 4% to 5% of the patients’ mutation characteristics were consistent with the DNA mutation pattern caused by colistin. This study established a direct connection between microbes in the human body and genetic variation that drives cancer development (Pleguezuelos-Manzano et al., 2020).

*E. coli*-induced DSBs can be identified by the suspension fragmentation labeling *in situ* sequencing (sBLISS) method (a DSB capture method based on sequencing), and the action of colistin directly related to AT-rich sequences can be found by DREME (motif identification method) (Dziubańska-Kusibab et al., 2020). By predicting DNA shape parameters at the central position of all 4096 possible hexanucleotides and correlating them with the sequence enrichment ratio of hexanucleotides at DSB positions in PKS+ and PKS- *E. coli-*infected cells, the colicin-specific six nucleotide sequences AAAATTT \ AATTTT and AAAAAATTT were detected. They had the narrowest microgroove width and the largest negative electrostatic potential. The extreme characteristics of colicin destruction motif (CDM)-related sequences were confirmed. Theoretical single base substitution (SBS) markers based on the frequency of trinucleotide sequences were deduced. The main characteristics of SBSA markers were T> C mutations at ATA, ATT and TTT trinucleotides and T> G mutations at TTT. Moreover, the prediction of the total mutational load in CRC cohort WGS by this marker is significantly correlated with the proportion of mutated CDM, indicating that the trinucleotide marker of SBSA can represent colistin-specific mutations (Dziubańska-Kusibab et al., 2020).

X (PH2A.x) is a known marker of DNA double-strand breaks and activation of the DNA damage response. Pancreatic cell damage was detected by measuring phosphorylated γH2A. In nonmalignant pancreatic cells, *G. adiacens* H1, *E. faecalis* C1 and *Klebsiella oxytoca* H1 strains caused strong PH2A.x (Halimi et al., 2021). In pancreatic malignant cell lines such as Capan-2 and AsPC-1, PH2A.x increased. Generally, the strongest inducer of PH2A.x is *E. cloacae* isolated from an intraductal papillary mucinous neoplasm (IPMN)-cancer case. Application of penicillin−streptomycin in the early stage of coculture of bacteria and cells could completely prevent DNA damage, indicating that IPMN-encapsulated bacteria could cause significant DNA damage to healthy pancreatic cells in the early and late stages of cancer (Halimi et al., 2021).

#### 4.3.2 Bacterial induction of epigenetic changes in the host genome

Epigenetics is a chemical marker system that annotates genetic information by affecting protein binding and chromatin structure and causing changes in gene expression. DNA methylation, histone modifications, noncoding RNAs and RNA splicing factors can change the chromatin and transcriptional program of host cells. These heritable changes, which alter gene expression but do not affect the DNA sequence, are called heterologous variations. Aberrant modifications of gene regulatory regions lead to abnormal cell development and accumulation of damaged DNA (Roadmap Epigenomics Consortium et al., 2015; Skvortsova et al., 2018). The intestinal tract has the most abundant microorganisms. Intestinal cancer microorganisms are related to the carcinogenesis of intestinal mucosal cells. By comparing DNA methylation in colonic crypt intestinal epithelial cells of germ-free mice and conventional mice through whole genome bisulfite sequencing (WGBS), global methylation levels were found to be significantly reduced in conventional mouse samples. A set of “sentinel genes” activated by demethylation in conventional mice that were responsible for normal regeneration of intestinal mucosa in the healthy gut were identified. This microbiome-dependent demethylation corresponded to the increase in the expression and activity of the DNA demethylases Tet3 and Dnmt1 in the intestinal epithelial cells of conventional mouse colon crypts, in which the deletion of specific Tet3 led to an increase in overall DNA methylation, including sensitive areas of the microbiome. In a mouse model of chemically induced sodium dextran sulfate (DSS) colitis, the phenomenon of DNA methylation reduction in intestinal mucosal cells caused by intestinal microorganisms was found. The decrease in DNA methylation was related to the activation of many genes related to intestinal inflammation and cancer, suggesting that intestinal bacteria might reprogram the DNA activity of intestinal mucosal cells (Ansari et al., 2020). Nitric oxide molecules that nitrosylated the host ALG1 protein were found by tracking the nitric oxide secreted by the model organism nematode *in vivo*. Excessive nitric oxide affected the normal development of nematodes, resulting in deformity. Notably, ALG1 is a highly conserved protein evolutionarily from nematodes to humans. In mammals, when commensal microorganisms produce too much nitric oxide, this protein may also be affected by the same S-nitrosylation modification, possibly causing developmental problems as well (Seth et al., 2019).

Posttranslational modification of histones is another central mechanism by which microorganisms regulate host chromatin. These types of modifications are typically not directly targeted to DNA but are covalently added to lysine residues in histone tails to affect chromatin conformation and gene expression. HDAC3, a histone deacetylase, is highly expressed in the intestinal epithelium and is sensitive to microbial signals. Cell-specific deletion of HDAC3 in intestinal mucosa increased H3K9 acetylation and gene expression, altered Paneth cell homeostasis and intestinal barrier function, and worsened the response to DSS-induced intestinal inflammation. Importantly, abnormalities in HDAC3 deletion were evident only in conventional mice but not in germ-free mice, suggesting tight communication between gut bacteria and the host genome (Alenghat et al., 2013). There are many microRNAs from small intestinal epithelial cells in mouse and human feces. These fecal microRNAs can enter bacteria, such as *F. nucleatum* and *E. coli*, and specifically regulate the transcription of bacterial genes to affect bacterial growth. If Dicer, the enzyme responsible for microRNA processing, is specifically knocked down in these two types of cells, fecal microRNA is reduced (Liu et al., 2016). Conversely, microRNAs from bacteria in feces might also enter the genome of human cells and regulate gene stability, which is worth exploring. *F. nucleatum* promoted the proliferation of CRC cells and the development of cancer by activating the Toll-like receptor signaling pathway in intestinal mucosal cells to nuclear factor-κB and upregulating the expression of microrNA-21. Bacteria can regulate the expression of the human host genome, thereby affecting cell functions (Yang et al., 2017).

#### 4.3.3 Bacterial induction of point mutations in the host genome

Bacteria can destabilize the human genome, leading to events such as base substitutions and copy number variations. *Staphylococcus aureus* evolved into methicillin-resistant *Staphylococcus aureus* (MRSA) through gene mutation to resist the elimination of human immune cells and the killing effect of antibiotics. The prevalence of antibiotic-resistant *staphylococcal* infections is increasing, and human cells are fighting back in kind. By sequencing the whole exons of 32 patients who had persistent MRSA infections and 32 patients who were able to clear the infection from their blood, approximately 62% of patients who cleared MRSA infection were found to have had obvious genetic variation, while only 9% of patients had persistent infection. This mutation was located in the DNMT3A region of chromosome 2P (Mba Medie et al., 2019). The P53 gene was the first and most famous tumor suppressor gene discovered by human beings. The main function of the P53 protein encoded by the P53 gene is to repair DNA and prevent cell carcinogenesis (Derech-Haim et al., 2020). P53 was introduced into a mouse cancer model, and two opposite effects were observed: inhibiting the occurrence of proximal CRC but promoting the occurrence of distal CRC. Microbial abundance gradually increases from top to bottom along the intestinal tract; that is, there are fewer microorganisms in the proximal intestinal tract and more microorganisms in the distal intestinal tract. In organ-like tissues of the proximal intestine and sterile ileum, P53 could better inhibit WNT gene expression and cancer occurrence (Kadosh et al., 2020). When the intestinal microbiota in the mouse model was eliminated with antibiotics, the abnormal development of the colon and ileum was eliminated, and the activation of the WNT gene was also reduced. These results suggested that the gut microbiota could counter the p53-mediated inhibition of WNT, thereby promoting the occurrence of distal bowel cancer (Kadosh et al., 2020). Bacteria affect the stability of the human genome and affect the expression of host genes.

### 4.4 Bacterial DNA induction of carcinogenesis

The DNA released by bacteria travels around human cells and then into the nucleus. Bacteria-induced changes, such as DNA strand breaks and genomic instability, provide an opportunity for bacterial DNA to integrate into the host genome. The latest research on bacterial DNA integration into the human genome is described in Part 3. Approximately one-third of healthy genomes contained bacterial DNA sequences, and even more were found in cancer cells. Bacterial genes that transfer into the human genome may initiate the transformation of healthy cells into cancer cells by stimulating protooncogenes or inhibiting oncogenes (Riley et al., 2013). Proto-oncogenes are present in normal cells but are not activated. Under the action of various environmental or genetic factors, the structure of proto-oncogenes is changed and activated into oncogenes, leading to excessive or continuous growth signals (Bolen and Veillette, 1989). Tumor suppressor genes can inhibit cell growth. They exist in normal cells and play a negative role in regulating cell proliferation. Their inactivation or deletion can lead to the survival of mutant cells and promote cancer progression (Marshall, 1991). For example, the CDKN2A, CDH1 and RUNX3 genes found in precancerous lesions of *H. pylori*-infected patients and gastric cancer patients could inactivate tumor suppressor genes. Gastric cancer-related mutations were identified in TP53, CDH1, ARIDIA and RHOA genes (Pandey et al., 2018; Capparelli and Iannelli, 2022). The genomes of cancer cells may be more receptive to bacterial genomes than normal cells.

**Figure 2:**
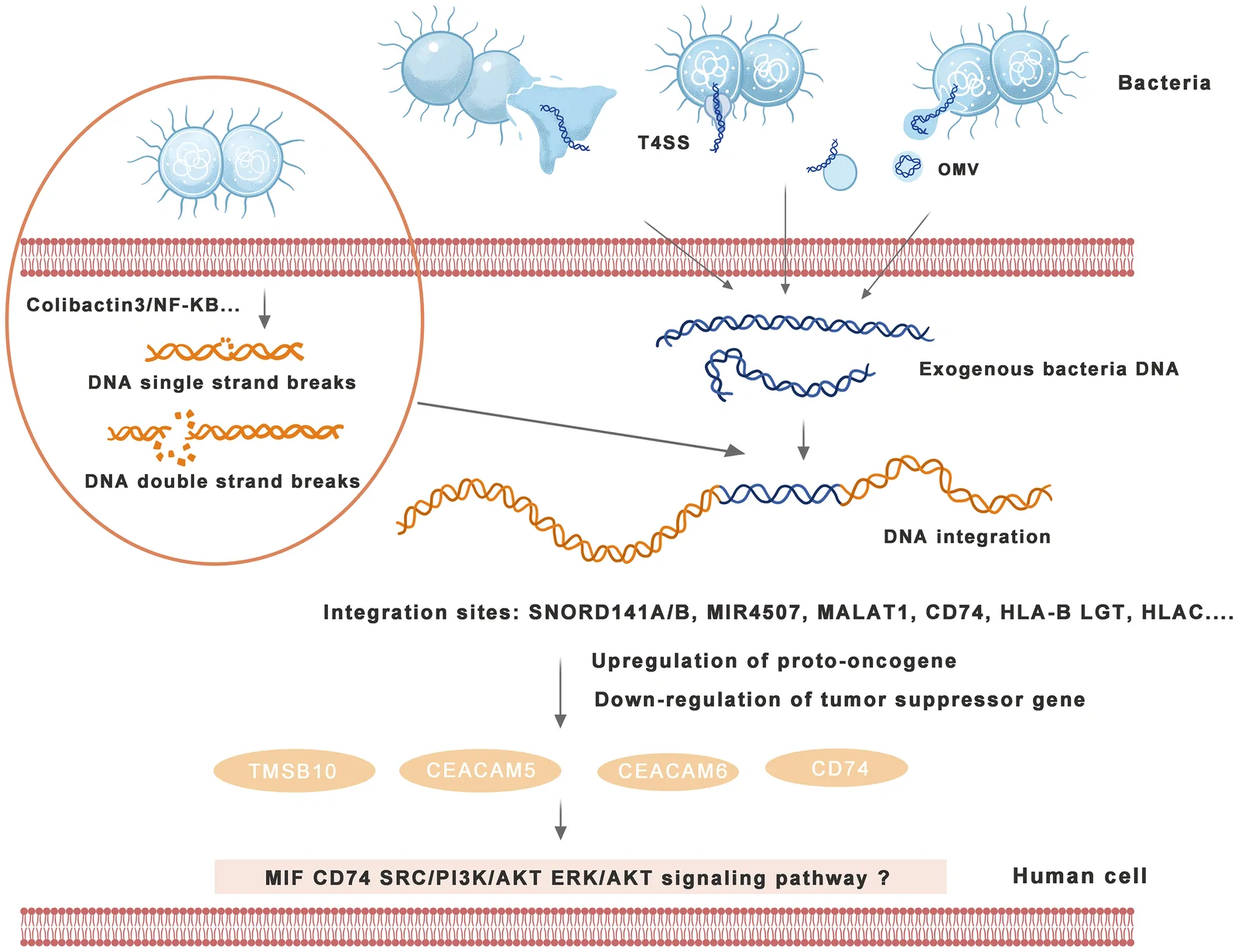
Mechanism of bacterial DNA integration into the human genome and induction of cancers. Bacteria excrete DNA through direct exclusion, outer membrane vesicle (OMV) excretion, type IV secretion system (T4SS), etc., and human cells receive exogenous DNA with transformation ability. Bacteria can induce human DNA single-strand breaks (SSBs) or double-strand breaks (DSBs) through a variety of signaling pathways, providing conditions for DNA integration into the human genome. Human cells integrate exogenous bacterial DNA into the exon or intron region and activate carcinogenic signaling pathways and induce tumors by enhancing proto-oncogene expression or weakening tumor suppressor gene expression in the human genome.

The mechanism of bacterial DNA integration into the human genome and induction of cancers are shown in **Figure 2**.

## 5 Three sources of bacterial DNA in human cancer

### 5.1 Bacterial DNA around human tissues

There are approximately 3.8×10^13^ bacteria in the 70 kg standard weight reference human body, most of which exist in the human digestive tract (Sender et al., 2016). In addition, other organs communicating with the outside world, such as the lung, colorectal and bladder, may have a large number of bacteria around them (Jin et al., 2019; Mansour et al., 2020; Liu et al., 2021; Bell et al., 2022). At present, many microorganisms, including *Helicobacter pylori* and *Escherichia coli*, have been found to affect the homeostasis of the genome in different ways and even directly damage DNA groups (Tjalsma et al., 2012; Sears and Garrett, 2014; Dejea et al., 2018). Genome instability provides an opportunity for the integration of exogenous bacterial DNA. *Escherichia coli*, *Bacillus subtilis*, etc., can establish natural competence through their internal regulatory mechanisms and can “directly” ingest foreign DNA in the natural environment (Torres et al., 2019; Sun et al., 2020). With the discovery of DNA molecules and competent cells with transformation activity, the role of natural transformation in horizontal gene transfer has become a focus of research. So-called natural transformation is a type of gene transfer method in which naked DNA molecules interact with natural competent cells without any medium. Natural transformation can occur between bacteria or between bacteria and other eukaryotes (Durieux et al., 2019; Simpson et al., 2019; Torres et al., 2019; Sun et al., 2020; Bonifácio et al., 2021). In recent years, the phenomenon that bacterial cells can actively secrete DNA that has transformation activity into the environment has been observed (Durieux et al., 2019; Simpson et al., 2019). If the bacterial cells around human tissue cells can actively secrete DNA with transforming activity and human cells can actively take up the surrounding bacterial DNA, then the bacteria around human tissue may be a major source of human genes. Human cells integrate ingested exogenous DNA into the genome through mechanisms such as DNA insertion or replacement, and the resulting changes in the expression of cancer-promoting or cancer-suppressor genes may be a mechanism of bacteria-induced cancers.

### 5.2 Free bacterial DNA in bacteremia or septicemia

Bacterial infection into the blood circulation is called bacteremia (Fontana et al., 2021). Pathogenic bacteria invade the blood circulation, grow and reproduce in the blood, and produce toxins. Acute systemic infection is called septicemia (Mirouse et al., 2020). Cell-free circulating DNA (cfDNA) refers to the DNA fragments that come from apoptosis or necrosis of cells and are free from outside the cells and widely exist in human serum and plasma. The cfDNA in the blood contains genomes from all types of tissues and cells in the entire body and theoretically also contains genomes of all types of viruses and microorganisms (Goggin et al., 2020; Xiao et al., 2021).

Stephen R. Quake and his team of Stanford University analyzed the cfDNA sequencing data of 1351 samples, extracted nonhuman sequences from them, and found that some of them could match the known bacterial genomes after assembly, indicating that there was free bacterial DNA in blood (Kowarsky et al., 2017). Based on this finding, many researchers have begun to try to detect bacteria with free bacterial DNA. Professor Samuel Yang’s team detected bacterial DNA in the blood of 350 patients with a sepsis alarm, and the results showed that 93.7% of them were consistent with blood culture, verifying that there was free bacterial DNA in the blood (Blauwkamp et al., 2019).

Human cells need a blood supply to grow continuously. At this time, bacterial DNA can reach tissue cells through the blood supply. When tissue cells come into contact with bacteria or bacterial DNA, the genes in foreign cells are likely to enter one or more tissue cells. Although there are unique enzymes in the human body that can prevent foreign genes from entering, some studies have shown that some foreign genes are similar in appearance to human cellular DNA. When genes are actively inserted into the genome of tissue cells, tissue cells will mistake foreign genes for their own genes and start reading them and using them to synthesize protein. Different bacterial DNA may have different affinities to different tissues and cells, which is related to the tumor specificity of bacterial DNA. Professor Rob Knight’s team from the University of California San Diego used the random gradient enhanced ML model to analyze the acellular microbial DNA in plasma (Poore et al., 2020) and found that acellular microbial DNA has a high degree of discrimination for various cancer types, such as prostate cancer, lung cancer and melanoma.

Therefore, we hypothesize that the free bacterial DNA in the blood may be a source of bacterial genes in human tumors, and they likely participate in the occurrence of breast cancer and other tissue tumors that are different from the external environment. The specific situation deserves further study.

### 5.3 Endogenous bacterial DNA in the human genome

Based on the first two hypotheses, human cells collect genes released from bacteria and take them for their own use. If the foreign genes are well adapted, human cells will pass on foreign genes to offspring during proliferation, so once a cell carries bacterial genes, it can be passed on to offspring, which is called vertical gene transfer. At present, the most typical example is mitochondria in cells (Ku et al., 2015a; Ku et al., 2015b).

Endosymbiosis holds that approximately 1.7 billion years ago, an aerobic bacterium called gram-negative bacteria invaded the host cell, but the host cell did not engulf the bacteria. Instead, the host cell provided a steady stream of nutrients for the bacteria, and the bacteria efficiently decomposed the nutrients into energy and provided it to the host. The cooperation between the two greatly enhanced their viability and gradually occupied a dominant position in the biological competition, and the bacteria evolved into mitochondria today after hundreds of millions of years (Dziubańska-Kusibab et al., 2020; Halimi et al., 2021). The relevant evidence is as follows: ① mitochondria have genetic material independent of the nucleus; ② mitochondria are one of a few structures with double membranes in cells, and the inner membrane of mitochondria is similar to the bacterial membrane; mitochondrial DNA is circular, similar to that of some bacteria, and the translation process of mitochondrial protein is more similar to that of some bacteria; and ④ mitochondria proliferate in a similar way to bacteria. In 2020, mitochondria were shown to originate from *Proteus alpha* by phylogenetic analysis based on systematic species sampling (Fan et al., 2020). Combining the two viewpoints that bacterial DNA can exist after entering human cells and that cancers have familial inheritance, we present the hypothesis that cancer-causing bacterial DNA can be inherited after being integrated into the human genome, which will lead to hereditary tumors in people with high susceptibility.

**Figure 3:**
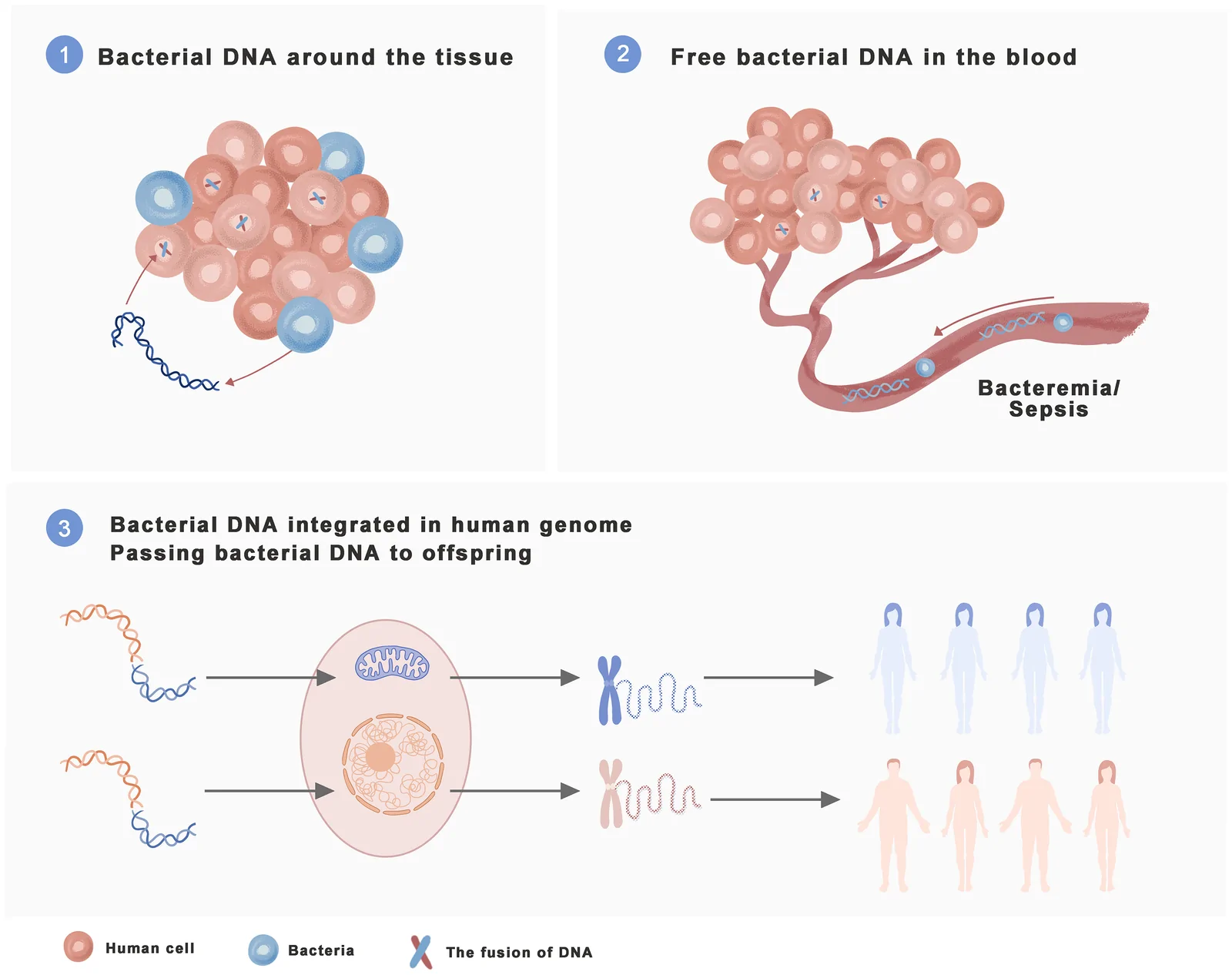
Three Sources of bacterial DNA in human cancers. ① Organs that are direct contact with the outside world, such as the colorectal area and lung, are full of bacteria, and tissue cells are almost bathed in bacteria. The DNA excreted by the bacteria surrounding the tissue can enter receptive human cells, resulting in gene integration. ② Breast cancer, bone cancer and other organs that are not exposed to the outside world usually cannot come into direct contact with a large number of bacteria. However, when the body is infected with bacteremia, septicemia, etc., they can come into contact with bacteria and their DNA in the blood circulation. The greater the demand for blood supply, the greater the chance of contact. Human cells come into contact with and absorb bacterial DNA, resulting in gene integration. When bacterial DNA is integrated with the human genome, it will persist in human cells without incident. Genes are vertically inherited and will be passed on to future generations. This inheritance may be the cause of endogenous bacterial DNA in the human body.

Three sources of bacterial DNA in human cancers are shown in **Figure 3**.

## 6 Conclusion

The existence of bacterial DNA in tumor tissue and its surroundings has been confirmed by a series of molecular experiments and biochemical analysis methods. We hypothesized three sources of bacterial DNA fragments: bacterial DNA transferred horizontally by bacteria near human cells, bacterial DNA remaining in human blood after bacterial infection, and bacterial DNA passed down from progenitors. For bacterial DNA to be expressed in the human body, it may undergo the following processes: it is released from bacteria, crosses the cell membrane of human cells, crosses the nucleus, and integrates into the human genome. The damage caused by bacteria to human DNA, such as inducing DNA breaks, regulating gene expression by epigenetic modifications, and causing genome instability, can facilitate the integration of bacterial DNA into the human genome. Human cells fail to remove and repair bacterial DNA in time, resulting in the persistence of bacterial DNA in the body. Changes in the host genome by bacterial DNA may activate proto-oncogenes and suppress tumor suppressor genes in human cells, causing the host cells to become cancerous.
