# Correction: Bacterial DNA involvement in carcinogenesis

**DOI:** 10.3389/fcimb.2026.2006085

**Published:** 2026-10-06

**Authors:** Han Shuwen

**Affiliations:** 1 Huzhou Central Hospital, Affiliated Central Hospital Huzhou University, Huzhou, China; 2 Graduate School of Medical College of Zhejiang University, Hangzhou, China; 3 Key Laboratory of Multiomics Research and Clinical Transformation of Digestive Cancer, Huzhou, China

**Keywords:** bacterial DNA, human genome, gene integration, cancerogenesis, hypothesis

In the published article, author Wang Yangyanqiu was erroneously included as an author in the published article. The correct author list reads:

Han Shuwen ^1,2,3*^

“^1^Huzhou Central Hospital, Affiliated Central Hospital Huzhou University, Huzhou, China.

^2^Graduate School of Medical College of Zhejiang University, Hangzhou, China.

^3^Key Laboratory of Multiomics Research and Clinical Transformation of Digestive Cancer, Huzhou, China.”

The **Author Contributions** Statement has been corrected to read:

“HS: design, writing, and approval of the manuscript.”

The **Acknowledgements** section of the review has been supplemented with the statement: “The author acknowledges Yangyanqiu Wang for his comments and suggestions during the preparation of this work.”

The original version of this article has been updated.

